# Vibration Signal-Based Non-Destructive Evaluation on Dynamic Vibration Characteristics and Anti-Disturbance Performance of Underground Coal Mine Anchor Cables

**DOI:** 10.3390/s26196034

**Published:** 2026-09-23

**Authors:** Jianju Ren, Bao Zhang, Xinyi Liu, Wenlong Zhang, Yaqi Zhou, Ruitao Zhou

**Affiliations:** 1 School of Resource Engineering, Xi’an University of Architecture and Technology, Xi’an 710055, China; rjj18049300680@xauat.edu.cn (J.R.); zhangbao122320@xauat.edu.cn (B.Z.); 2 School of Smart City Engineering, Qingdao Huanghai University, Qingdao 266427, China; 17861329796@163.com (X.L.); 17860759953@163.com (Y.Z.); 3 School of Information Science and Engineering, Qingdao Huanghai University, Qingdao 266427, China; zhourt@qdhhc.edu.cn; 4 Beijing Key Laboratory of Super Intelligent Technology for Urban Architecture, Beijing University of Civil Engineering and Architecture, Beijing 102616, China

**Keywords:** non-destructive testing, dynamic response, anti-interference ability, stress wave, stress disturbance

## Abstract

The behavior of anchor cables in response to stress disturbances directly influences the effectiveness and interference resistance of their support system. This study developed and executed a non-destructive testing method to evaluate and compare the dynamic responses of anchor cables made from various materials under stress disturbances. The methodology utilized two vibration sensors in conjunction with a single data acquisition instrument. The vibration sensors were responsible for capturing the vibrational signals of the anchor cable, while the acquisition instrument facilitated synchronous data collection. The anti-interference ability was compared by the collected vibration signals of different anchor cables under the influence of the same stress disturbance. The results showed that the IREA anchor cable exhibited lower vibration intensity and stronger anti-interference ability than the ordinary anchor cable under the same stress disturbance, which could be quantitatively compared by the proposed method. Across 16 disturbance events, the vibration amplitude ratio of the ordinary anchor cable to the IREA anchor cable ranged from 1.3 to 5.3, with an average ratio of 2.45. The method was conceptually straightforward and easily implementable, and the field measurement data demonstrated promising results.

## 1. Introduction

In the field of geotechnical engineering [1,2], anchor cables are used in various situations to provide strong support [3], ensuring the integrity and safety of the surrounding rock [4]. The principle of anchor cable support is very simple, the main body is firmly combined with the surrounding rock through deep anchoring sections [5], and the exposed sections are fixed with bolts and trays to compress and stabilize the surrounding rock [6,7]. In recent years, with the development of technologies and materials, many types of excellent anchor cables have emerged [8], which usually have better tensile strength [9] or shear strength [10], elongation, energy absorption performance [11], creep behavior [12], or added certain extensions [13] and constant resistance devices [14]. The application of anchor cables has always improved the safety of geotechnical engineering such as roadways/gateways [15], tunnels [16], slopes [17], and other underground excavations [18]. However, the testing methods for certain performance indicators of anchor cables have always been a problem worthy of in-depth research [19].

The inspection indicators of anchor cables usually included breaking strength [20], yield strength [21], elongation [22], energy absorption [23], anchoring capacity, stiffness [24,25], nonlinear dynamic response or stability properties [26], etc., but these are usually completed in the laboratory [27]. For example, after inventing a new type of anchor cable, scholars conducted pull-out tests in the laboratory to prove that it has higher breaking strength [28]. Some scholars have invented devices that enhance the elongation of anchor cables while still ensuring support resistance, and have tested and verified their effect [29]. Researchers also set anchor cables/bolts in the surrounding rock to compare the overall anti-damage performance of the surrounding rock and support body with or without anchor cables/bolts [30]. Scholars have also conducted quantitative experiments on the impact energy absorption capacity of different anchor cables using the same stress disturbance in the laboratory [31]. For the evaluation and quantitative monitoring methods, vibration monitoring was widely and reliably applied in many industries and scenarios [32,33], and also suitable as a non-destructive monitoring method in coal mines [34,35].

However, it must be pointed out that there was a gap between the laboratory conditions and the actual conditions on site [36]. For example, the energy impact in the laboratory was difficult to reach the level of the site, and each impact for different cables could not be guaranteed to be the same [37,38]. In terms of on-site comparative testing, some researchers have compared different support structures constructed in different construction sections [39,40,41]. However, the conditions of different construction sections were inherently inconsistent, which bring the trouble of inconsistent scales to the comparison. On the on-site scale, there were few methods available to quantitatively compare the support performance of different anchor cable supports. This lack of quantitative comparison methods was a bottleneck hindering the full and effective implementation of new support structures. Although some anchor cables had good performance and have been validated in the laboratory or simulation, there was still practically no reasonable method to prove and demonstrate their effectiveness in field applications, which may reduce their usage and application effect on site.

Therefore, this study aims to propose a convenient non-destructive testing measurement method for quantitatively comparing the effects of different anchor cables on site, especially under the same stress disturbance. After careful consideration and testing, a method has been developed that utilizes vibration signals to provide feedback on the degree of dynamic disturbance impact. This method is effective when there is a natural seismic source in the underground coal mine gateway, and the impact disturbance from the seismic source on adjacent anchor cables is consistent. By using a synchronized vibration signal acquisition system, it is possible to monitor and compare the dynamic responses of multiple anchor cables simultaneously.

## 2. Method

The overall procedure of the proposed non-destructive testing method is illustrated in Figure 1. The method mainly consists of four steps: applying the same external stress disturbance to the tested anchor cables, synchronously collecting the corresponding vibration signals, extracting the vibration response for each disturbance event, and quantitatively comparing the vibration responses of different anchor cables. Under the same stress disturbance and measurement conditions, the vibration response characteristics of the ordinary anchor cable and the IREA anchor cable can be compared based on the recorded vibration amplitude and statistical parameters.

A gateway of an underground coal mine in Inner Mongolia was selected as the monitoring site. There were two kinds of anchor cables in the gateway construction, one was the ordinary anchor cable, and the other was the Isolation Resilient Energy Absorbing (IREA) anchor cable. IREA anchor cables were proposed by [42] and had the characteristics of customizable extension length, adjustable cable stiffness, and strong impact resistance. In the production process of steel bars, IREA anchor cables used special forging technology, which increased their elongation by nearly 6 times compared to ordinary anchor cables, while maintaining the ultimate bearing capacity unchanged.

There were some differences between ordinary anchor cables and IREA anchor cables in the forging process, elongation and yield strength, which provided an important basis for specific testing methods. If the performance difference between two types of anchor cables was small, assuming only different types of conventional anchor cables, and there was no significant difference in forging and manufacturing processes, it may be difficult to reflect the difference in measurement results during the testing process. Based on this, ordinary anchor cables and IREA anchor cables were selected for testing in the study to verify the feasibility of the adopted method. Due to the significant differences between these two types of anchor cables in the key aspects mentioned above, the test results could be more distinguishable, thereby effectively verifying whether the method could accurately capture the characteristic differences between different anchor cables, and providing reliable basis for subsequent related research and practical applications. The measurement method used in this study also provides an important basis for verifying the dynamic response and energy absorption capacity of IREA anchor cables. If the measurement and verification method is effective, it can be used as a reference for testing other new types of anchor cables in the future.

The chosen gateway was frequently affected by mining and has a unified source. In the field test, two vibration sensors were used, which were respectively arranged at the ends of the ordinary anchor cable and IREA anchor cable, and were installed by magnetic suction (the anchor cable body was steel strand, and the sensor mounting base had magnetic suction function). The WIFI wireless signal transmitter was connected behind the sensor by wired connection, and the WIFI signal was transmitted to the WIFI signal collector. In previous practical tests on the WIFI-based data transmission system, it was confirmed that the WIFI transmission method had a stable data transmission speed and a low data loss rate. Moreover, wireless transmission was more convenient for the layout and installation of mobile devices, which could improve the convenience of on-site testing. In summary, the whole acquisition system included a WIFI signal collector and two vibration sensors with WIFI conversion and transmission (WIFI wireless signal transmitter), as shown in Figure 2.

The model of the WIFI signal collector was KJD3.7-A (Xuzhou, Jiangsu, China), and the acquisition frequency of each channel was 1000 Hz, which could achieve the real-time acquisition, accurate signals, and storage of WIFI signals for subsequent analysis (previous study of [43] showed that the dominant frequency of underground vibration signals was mostly below 300 Hz. A sampling rate of 1000 Hz was approximately three times the highest frequency of the vibration signal, which conformed to the Nyquist theorem). The vibration sensor tape was GBC100W (Xuzhou, Jiangsu, China), with a range of 0~20 mm/s, a sensitivity calculated to be 1.2 V/(m/s) (corresponding to a 12~24 V output voltage range), and a frequency response of 5~1000 Hz. Calibration was strictly carried out by the manufacturer (Xuzhou Dongkong Instrument Co., Ltd., Jiangsu, China) on the vibration table in accordance with relevant regulations and meets factory requirements. In the scene of the gateway in the underground coal mine, the effective distance of WIFI signal transmission was up to 20~50 m, and it could also provide a guarantee for accurately evaluating the energy absorption capacity of anchor cables. If the sensor system in this study could quantitatively evaluate the anti-interference performance of anchor cables, similar parameters and methods could also be used in other application scenarios, which reflects the contribution of the system in measurement.

The site settings of the monitoring system were shown in Figure 2. From the actual-object photos, it could be observed that the system featured simplicity and convenience. Before the system signal acquisition, a hot spot was first created and two vibration sensors were connected through WIFI wireless technology. After it was confirmed that the vibration signal was properly connected, as indicated by the green indicator in the corresponding channel of the collector’s software, the data acquisition was initiated. In the actual collection, more than two hours of data every time were collected (multiple tests were designed and implemented), and then the data of the signal collector was imported into the computer through USB connection for subsequent analysis. The acquired vibration signals were subsequently analyzed based on the recorded amplitude data. For each identified stress disturbance event, the corresponding vibration response was extracted for statistical analysis, including mean amplitude, peak amplitude, standard deviation, and variance. These parameters were used to characterize and compare the dynamic response of the two types of anchor cables under the same stress disturbance.

## 3. Results

Overall comparison of monitoring results between ordinary anchor cable and IREA anchor cable is shown in Figure 3. The figure displayed a representative 20 min segment of data, selected from a larger dataset to illustrate the key differences in performance. The abscissa in Figure 3 represented the number of points, and since the sampling frequency was 1000 Hz, it could be easily calculated that the time corresponding to the number of points in the abscissa range was about 20 min. Here, “Points” denotes the sampling-point index of the recorded vibration signal. With a sampling frequency of 1000 Hz, each sampling point corresponds to 0.001 s, and the sampling-point index can be directly converted to physical time by dividing it by 1000. It could be seen from the figure that the dynamic response of the conventional anchor cable was greater than that of the IREA anchor cable from beginning to end. The vibration amplitude of IREA anchor cable was generally below 1300 mV, while the vibration amplitude of the ordinary anchor cable sometimes even exceeded 5000 mV. From the curve form, the change trend of the two kinds of anchor cables was consistent. When there was vibration source influence (the stress disturbance caused by actual mining activities on site was not always present, but rather raised after the mining experience), both kinds of anchor cables have corresponding responses (like the labeled stress disturbance stage in Figure 3), and when there was no vibration source influence (like the labeled non-stress disturbance stage in Figure 3), both kinds of anchor cables had no response. That was to say, the change trend and form of the two kinds of anchor cables were consistent (similar to the consistency of changes in anchor cables from different perspectives in [5]), which also confirmed the consistency of the source and the accuracy of monitoring. From the overall monitoring results, this measurement method was effective, which could preliminarily determine the reaction degree of different anchor cables under the influence of stress disturbance.

In order to more clearly determine and analyze the dynamic (vibration) response of the two kinds of anchor cables, the signal comparison in Figure 3 was split and amplified to see the specific impact of each stress disturbance on the two kinds of anchor cables, as shown in Figure 4. The figure mainly showed the dynamic (vibration) response and box line chart comparison results of eight stress disturbance cases (#1~#8). From the response of stress vibration signals of eight cases, the vibration responses of the ordinary anchor cable were generally larger than that of the IREA anchor cable. From the specific manifestation of the curve, the two kinds of anchor cables both had vibration responses when the stress disturbance came, and the state of vibration change was consistent, which once again confirmed the unity of the stress disturbance and the accuracy of monitoring.

Figure 5 presented the normal Q–Q inspection plots generated from three sets of experimental data for the ordinary anchor cable and IREA anchor cable respectively. These plots were used to assess the distribution characteristics of the measured data during dynamic response tests and their conformity to the normal distribution. By comparing the quantiles of the sample data with those of the theoretical normal distribution, the Q–Q plots visually showed whether the data met the normality assumption, thus providing a basis for the rationality of subsequent statistical analysis methods. The Q–Q plots were used to examine the normality assumption for the statistical analysis of the event-level response data. It was observed from the figure that the three sets of data points for the IREA anchor cable were more closely distributed around the reference line, with smaller deviations within the upper and lower quantile intervals. This indicated that its dynamic response measurements under varying test conditions had better normal distribution characteristics. This distribution pattern suggested that the IREA anchor cable had been less influenced by random noise and had higher stability during the measurement process when subjected to disturbed loading. In contrast, the Q–Q plot for the ordinary anchor cable showed significant deviations in both the high and low quantile regions, especially with pronounced dispersion in the tail areas, which reflected certain non-normal characteristics in its response data.

From a specific numerical perspective, the vibration responses caused by the disturbance to the ordinary anchor cable in the eight cases in Figure 6 reached 739.3 mV, 1070.6 mV, 633.9 mV, 709.0 mV, 1125.5 mV, 998.9 mV, 1390.7 mV, and 809.9 mV, respectively, and the vibration responses caused by the disturbance to the IREA anchor cable reached 383.8 mV, 463.1 mV, 303.4 mV, 226.5 mV, 372.0 mV, 268.6 mV, 260.2 mV, and 246.9 mV, respectively. According to the statistical analysis presented in the box plot, the vibration impact on the ordinary anchor cable was consistently higher than that on the IREA anchor cable, with some instances showing more than twice the impact. From the data distribution of the box line chart, the same cluster of data appeared after a period of data, and the distribution of these data was symmetrical, which may relate to the vibration uniformity of the steel strand material caused by the stress interference. This fact meant that the average interference caused to the ordinary anchor cable reached as high as 934.7 mV, while the value of the IREA anchor cable was only 315.6 mV. Based on the average of the eight cases, the signal response caused by the same interference to the ordinary anchor cable was about two to three times that of the IREA anchor cable.

The curve and statistical results showed that the monitoring method adopted was effective, which could clearly observe that the IREA anchor cable had a lower vibration response in case of stress disturbance, and could better digest the impact of the disturbance. If the same monitoring method was used for monitoring and statistical analysis of other different anchor cables, the anti-interference and energy absorption effect of anchor cables could also probably be quantitatively evaluated. Moreover, from the perspective of big data, the overall curve and the interference results of the eight cases could roughly prove the effectiveness of the method. It should be a new measurement method to judge the dynamic response and anti-interference ability of different materials or types of anchor cables by the response degree of tail vibration signal under the same stress disturbance.

## 4. Discussion

The specific numerical comparison of the response to stress disturbance should be more able to reflect the performances of different anchor cables. Therefore, the response means amplitudes and ratios of two kinds of anchor cables under the influence of 16 stress disturbance cases (including the above eight ones in Figure 5) were quantitatively counted and shown in Figure 7. It was obvious from the figure that the mean amplitudes of the ordinary anchor cable when disturbed were generally greater than that of the IREA anchor cable, which was the situation in all 16 disturbance cases. According to the analysis of data statistics, the impact of 16 disturbance cases on the ordinary anchor cable was generally significant. Specifically, the effects caused by these interference factors exhibited a large fluctuation range in numerical values, with the largest interference effect exceeding 1400 mV, and even smaller interference effects maintaining values above 400 mV. In sharp contrast, the impact of the same 16 disturbance cases on the IREA anchor cable was relatively weaker. When the IREA anchor cable was disturbed, the small interference effect value was as low as about 200 mV, and even the maximum interference effect value was only slightly over 400 mV.

In terms of the specific proportion (ordinary vs. IREA), the largest was 5.3 times and the smallest was 1.3 times. According to the statistics, the mean ratio of 16 disturbance cases was 2.45. To further quantify the difference at the disturbance-event level, the response ratio of the ordinary anchor cable to the IREA anchor cable was calculated for each of the 16 paired disturbance events. The logarithm of the event-wise response ratio was then analyzed using a paired comparison framework. The normality of the log-transformed ratios was confirmed by the Shapiro–Wilk test (W = 0.959, *p* = 0.651). The analysis showed a significant difference between the two anchor cables, with a mean log-response ratio corresponding to a geometric mean ratio of 2.286 (95% CI: 1.875–2.788), t(15) = 8.886, *p* = 2.31 × 10^−7^, and Cohen’s dz = 2.221. The event-level statistical analysis further confirmed that the vibration response of the IREA anchor cable was lower than that of the ordinary anchor cable under the same disturbance conditions. The interference ability test method of different types of anchor cables under the same stress disturbance proposed in this study was relatively reasonable. In the process of coal mining, it was inevitable to cause mining disturbances, and the vibrations generated by these disturbances actually created natural favorable conditions for this testing method. From the analysis of the test results, the IREA anchor cable exhibited a lower dynamic response to the same stress disturbance than the ordinary anchor cable did. This result indicated that the IREA anchor cable had stronger anti-interference ability under the tested conditions. However, the measured vibration response was an indirect indicator and could not directly quantify the mechanical energy absorption or support the performance of the anchor cable.

Figure 8 showed the statistical results of the dynamic response standard deviation of the ordinary anchor cable and the IREA anchor cable, which were obtained from 25 sets of experimental data under the same testing conditions. The dispersion degree of the two types of anchor cables was analyzed by combining bar charts with violin plots. The standard deviation, an important statistical indicator for measuring the amplitude and stability of data fluctuations, directly reflected the sensitivity of the measurement system to random disturbances. From the bar chart results, it was evident that the standard deviation of the IREA anchor cable was significantly lower than that of the ordinary anchor cable in the vast majority of experimental events, indicating that the fluctuation amplitude of its dynamic response signal was smaller and the measurement results were more concentrated. The violin plot further revealed the overall shape of the standard deviation distribution. In it, the contour of the IREA anchor cable’s violin was narrower, the median position was lower, and the 25–75% quartile range was significantly reduced, indicating that its standard deviation distribution had good concentration and consistency. In contrast, the violin plot of the ordinary anchor cable showed a wider distribution range and was accompanied by certain long-tail characteristics, indicating a significant increase in dynamic response fluctuations under some experimental conditions. This phenomenon indicated that the ordinary anchor cable had limited ability to suppress environmental noise and external disturbances in complex working conditions in underground coal mines, which could easily lead to unstable measurement results. Overall, the figure indicated from the perspective of standard deviation statistical characteristics that the IREA anchor cable had better stability and anti-interference ability in dynamic response detection.

Figure 9 presented the statistical results of the dynamic response variance of the ordinary anchor cable and the IREA anchor cable under 25 experimental conditions, and visualized their discrete characteristics by combining bar charts and violin plots. As the square of the standard deviation, variance could more sensitively reflect the impact of extreme fluctuations on the overall data stability and was an important indicator for evaluating the anti-interference performance of a system. From the figure, it was observable that the overall variance level of the IREA anchor cable was significantly lower than that of the ordinary anchor cable, and the variation amplitude was relatively small across different experimental events. This indicated that the signal energy fluctuations of the IREA anchor cable were effectively suppressed during the dynamic response testing, and the system output was more stable. In the violin chart, the distribution pattern of the IREA anchor cable was relatively concentrated, with a low median position and a narrow quartile range, suggesting that their variance distribution had good repeatability and reliability. Conversely, the variance distribution of the ordinary anchor cable showed obvious discrete characteristics. In some experimental events, the variance value increased sharply, causing significant stretching of the violin plot. This phenomenon indicated that the ordinary anchor cable was more likely to generate large random fluctuations under dynamic loads and environmental disturbances, and the measurement signal contained more unstable components. From the perspectives of energy fluctuations and statistical stability, Figure 9 further demonstrated that the IREA anchor cable had significant advantages in anti-interference performance compared to ordinary anchor cables.

Figure 10 presented the bar chart results of the standard deviation ratio of the dynamic responses of the IREA and ordinary anchor cable under the same experimental conditions. This ratio was calculated with the ordinary anchor cable as the reference benchmark, quantifying the relative magnitude of the standard deviations of the two types of anchor cables to intuitively reflect the effectiveness of the IREA anchor cable in reducing response fluctuations. The bar chart showed that the standard deviation ratio was less than 1 in most experimental events, and, in some cases, it was significantly lower than 0.5. This indicated that the standard deviation of the IREA anchor cable was much smaller than that of the ordinary anchor cable under the same operating conditions. The result suggested that the IREA anchor cable could effectively suppress random fluctuations in the dynamic response and enhance the stability and consistency of measurement signals. In a few events, the ratio was slightly higher than 1, which might be associated with local differences in operating conditions or transient disturbances, but it did not alter the overall trend. From an engineering application perspective, a significant decrease in the standard deviation ratio implied that the IREA anchor cable had stronger noise resistance and higher measurement reliability in the complex underground coal mine environment. Figure 10 avoided the influence of absolute numerical differences through relative indicators, making the comparison of the performance of the two types of anchor cables more intuitive and convincing, and provided a quantitative basis for the application of IREA anchor cables in the field of dynamic monitoring.

Figure 11 presented the statistical results of the dynamic response variance ratio between the IREA and ordinary anchor cable and depicted the relative fluctuation energy levels of the two types of anchor cables in different experimental events through bar charts. The figure showed that the variance ratio of the IREA anchor cable was significantly less than 1 in most experimental events, and it was close to 0 in several events, indicating that the statistical fluctuation of its dynamic response was much lower than that of the ordinary anchor cable. This suggested that the IREA anchor cable had stronger suppression effects on external disturbances in terms of structural design or signal transmission mechanisms and could effectively reduce the impact of random interference on measurement results. Although there were relatively large variance ratios in individual experimental events, the overall distribution still showed a clear downward trend and no systematic reversal occurred. This indicated that the IREA anchor cable maintained stable anti-interference performance under most operating conditions. Based on the results, it could be concluded that the IREA anchor cable not only had a lower signal fluctuation amplitude but also exhibited greater stability in its dynamic response.

The quantitative evaluation of the support performance of support materials was important, and the actual field test was relatively few at present. Therefore, based on the consistency condition of stress disturbance in underground coal mine, this study proposed a measurement method to test the dynamic response and anti-interference ability of different anchor cables. The results showed that the vibration signal synchronous monitoring and evaluation method proposed in this study was effective. The response degrees of the ordinary anchor cable and IREA anchor cable to the same stress disturbance were obtained through synchronous monitoring. The overall signal trend results showed that the influence degree of the IREA anchor cable was less than that of the ordinary anchor cable. In addition, the study also focused on the analysis of the box diagram of the influence degree of eight disturbance cases on two kinds of anchor cables and quantified the effectiveness of the measurement method. And also, the response ratios of 16 stress disturbances to two kinds of anchor cables were counted, and the scientific degree of the measurement method was verified by relatively large data statistics.

This study chose ordinary anchor cables and IREA anchor cables as monitoring objects mainly because although both types of anchor cables use steel strands as the basic material, different forging processes were adopted in the design and manufacturing process. The IREA anchor cable has undergone special treatment, resulting in higher plasticity but lower brittleness, thus possessing greater elongation. This difference makes the IREA anchor cable significantly different in mechanical properties from the ordinary anchor cable, which is more conducive to comparing their characteristics. By comparing the performance of these two types of anchor cables, the advantages of the newly developed IREA anchor cable in practical applications can be better verified, especially in terms of dynamic response and energy absorption, providing an effective method for verifying the capabilities of other new anchor cables in the later stage.

This study proposed a method for measuring and evaluating anchor cables at the field scale, which was relatively rare in previous research. Previous studies had mostly focused on testing and experimenting with anchor cables in application scenarios such as slopes and tunnels at the laboratory scale [13,27]. Due to some limitations in laboratory conditions, natural seismic sources were usually not available and only artificial sources could be used. In contrast, the seismic source in underground coal mine tunnels was caused by mining, which was more suitable and stronger enough as a basis for comparing the performance of anchor cables and provided convenient conditions for the implementation of this research method. In addition, previous research had mainly focused on testing the mechanical properties such as elongation, tensile strength, failure strength, and bearing capacity of anchor rods or cables [14,20,23], with less involvement in vibration signal testing at the field scale. This was mainly because it was difficult to obtain convenient seismic sources on site. Therefore, this study had a certain degree of innovation in the selection of seismic sources and the processing and analysis of vibration signals at the field scale.

Overall, the quantitative results further demonstrated the difference in vibration response between the two types of anchor cables under the same stress disturbance. Across 16 disturbance events, the response amplitude ratio of the ordinary anchor cable to the IREA anchor cable ranged from 1.3 to 5.3, with an average ratio of 2.45. In addition, the standard deviation ratio of the IREA anchor cable to the ordinary anchor cable was lower than 1 in most disturbance events, and the variance ratio also showed an overall lower trend for the IREA anchor cable. These results indicate that the proposed method can distinguish the dynamic response characteristics and response stability of the two types of anchor cables under comparable disturbance conditions.

The proposed measurement in this study was analyzed to be effective, but there were also certain limitations. For example, although the same stress disturbance was applied to the distant part of the site, after all, the positions of the two anchor cables were slightly different, which may bring a slight impact. However, compared with the distance between the stress disturbance and the anchor cables (tens or even hundreds of meters), these could basically be ignored (the distance between the two anchor cables was only one to two meters). Nevertheless, differences in pre-tension, anchoring conditions, wave propagation paths, and sensor coupling may also affect the measured vibration response, and these factors should be considered in the further validation of the common-input assumption. Therefore, the observed differences should be interpreted as differences in the measured vibration response under the tested conditions rather than as direct measurements of the mechanical support performance of the anchor cables. Since only one ordinary anchor cable and one IREA anchor cable were monitored in the present field test, the repeated disturbance events should be regarded as repeated measurements rather than independent anchor-cable samples, and the generalizability of the results is therefore limited. In the follow-up of the study, it is necessary to expand the measurement range and simultaneously measure more anchor cables to more fully verify the accuracy of the measurement method.

## 5. Conclusions

This study proposed a non-destructive testing measurement method that utilized synchronous vibration signals to quantitatively assess the dynamic response and anti-interference capacity of anchor cables. The method leveraged the consistency of stress disturbances caused by mining activities in a gateway of an underground coal mine on different anchor cables, collecting and analyzing the dynamic response levels of the ordinary anchor cable and IREA anchor cable. Results indicated that both ordinary and IREA anchor cables exhibited consistent response curve variations under multiple stress disturbances, demonstrating the uniformity of stress disturbances. From both the overall curves and quantitative statistical results of case disturbances, the vibration sensor amplitudes of the ordinary anchor cable were generally larger than those of the IREA anchor cable, with the average ratio (ordinary vs. IREA) reaching 2.45. Across the 16 disturbance events, the amplitude ratio of the ordinary anchor cable to the IREA anchor cable ranged from 1.3 to 5.3. In addition, the standard deviation ratio and variance ratio of the IREA anchor cable to the ordinary anchor cable were lower than 1 in most disturbance events, indicating lower response fluctuations and greater response stability under comparable stress disturbances. These quantitative results further support the effectiveness of the proposed method in distinguishing the dynamic response characteristics of different anchor cables under the same stress disturbance. The results suggested that the IREA anchor cable experienced less impact under identical stress disturbance. In addition, from the standard deviation and variance results of the response vibration data, the IREA anchor cable was also superior to the ordinary anchor cable. The proposed non-destructive method required a shared stress disturbance source, and synchronous acquisition of vibration signals should be ensured from different anchor cables using identical sensors, thereby enabling comparable results. The developed measurement method provided substantial support for the quantitative comparison of the dynamic response characteristics and anti-interference capacity of different anchor cables under the same stress disturbance. However, only one ordinary anchor cable and one IREA anchor cable were monitored in the present field test, and the repeated disturbance events should therefore be regarded as repeated measurements rather than independent anchor-cable samples. The generalizability of the results is thus limited, and further field tests involving more anchor cables and different disturbance conditions are needed.

## Figures and Tables

**Figure 1:**
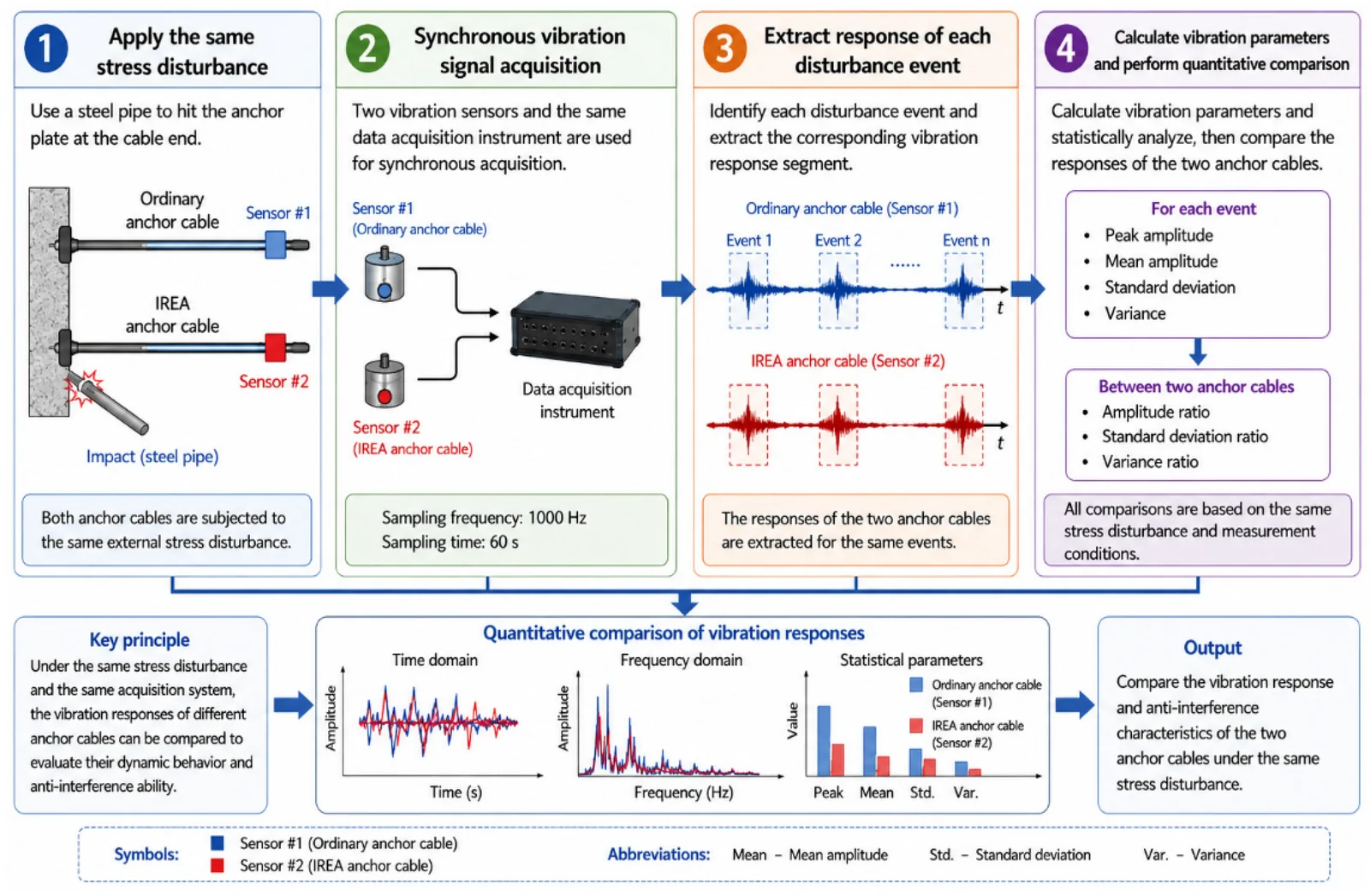
Flowchart of the proposed non-destructive testing method.

**Figure 2:**
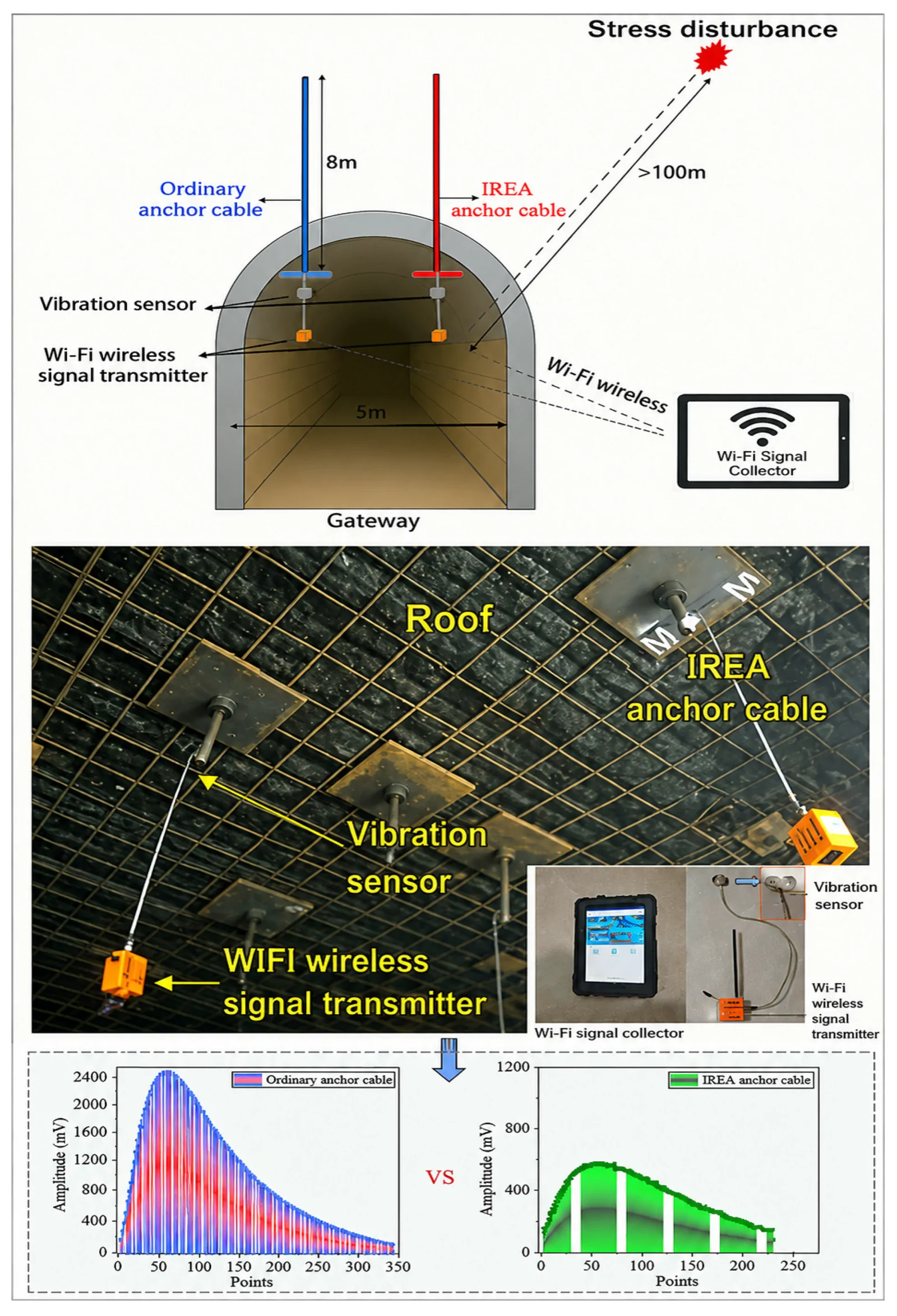
Overall layout and site settings of monitoring system.

**Figure 3:**
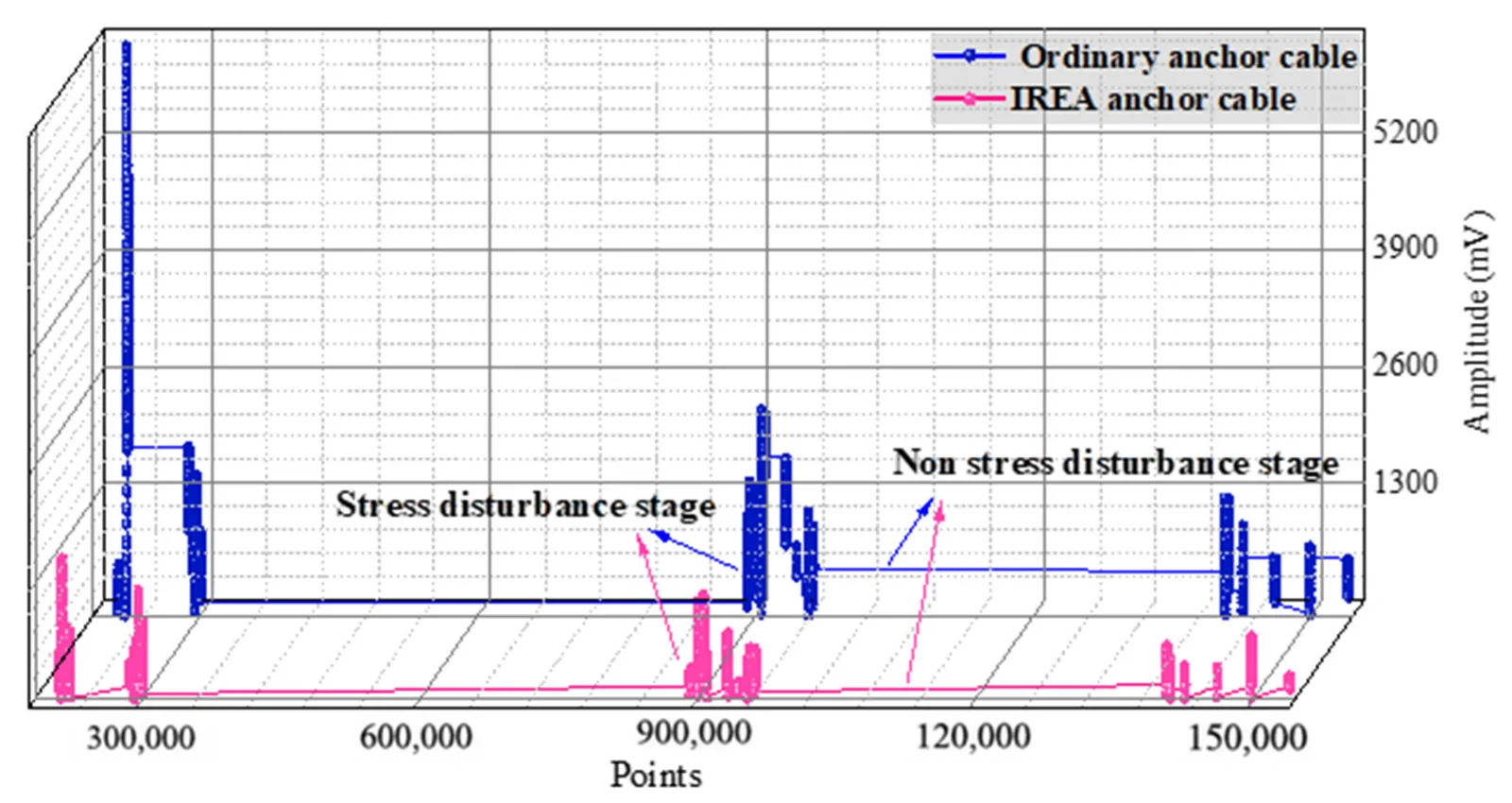
Overall comparison of monitoring results of ordinary anchor cable and IREA anchor cable.

**Figure 4:**
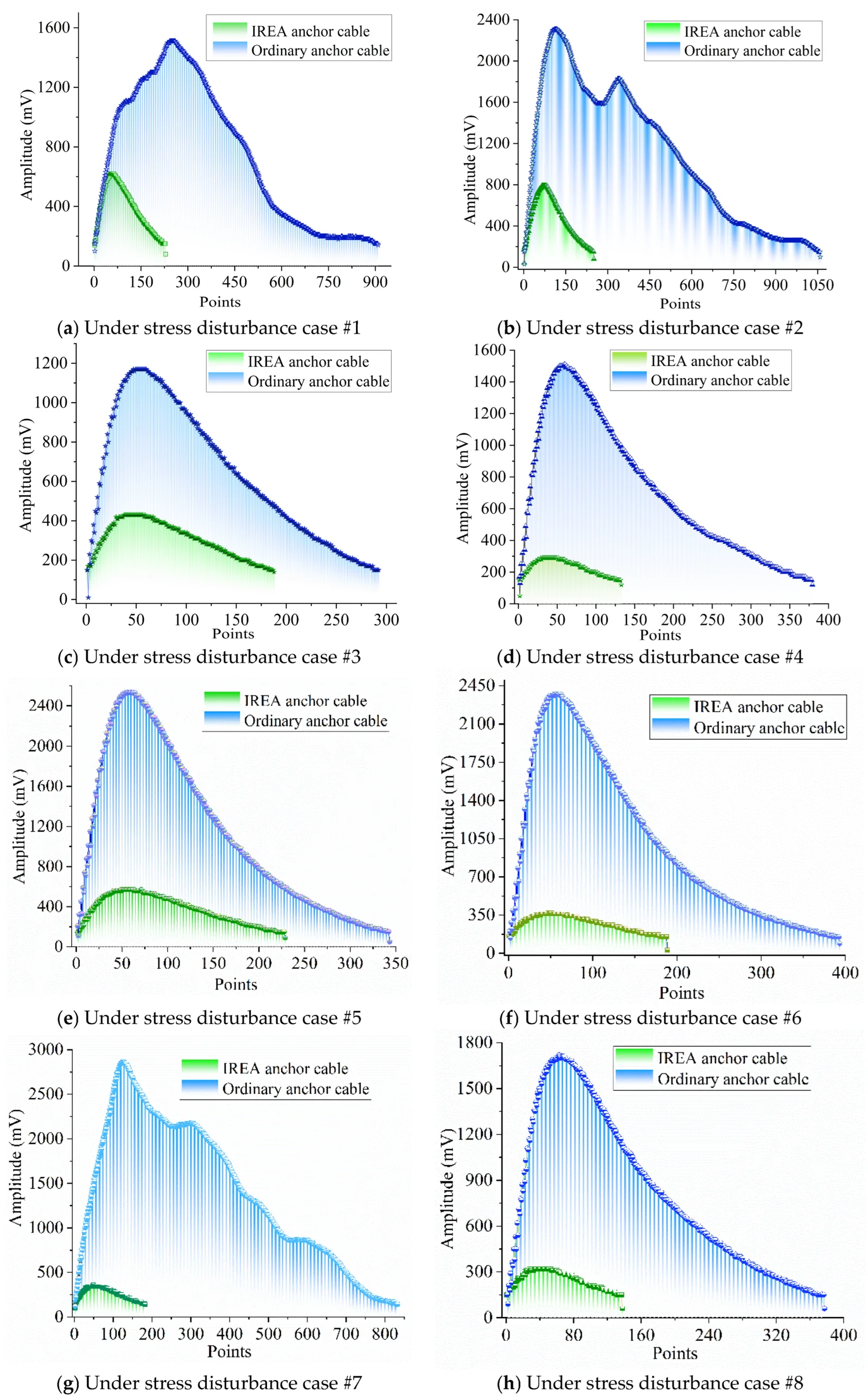
Dynamic (vibration) response comparison of stress disturbance cases #1~#8.

**Figure 5:**
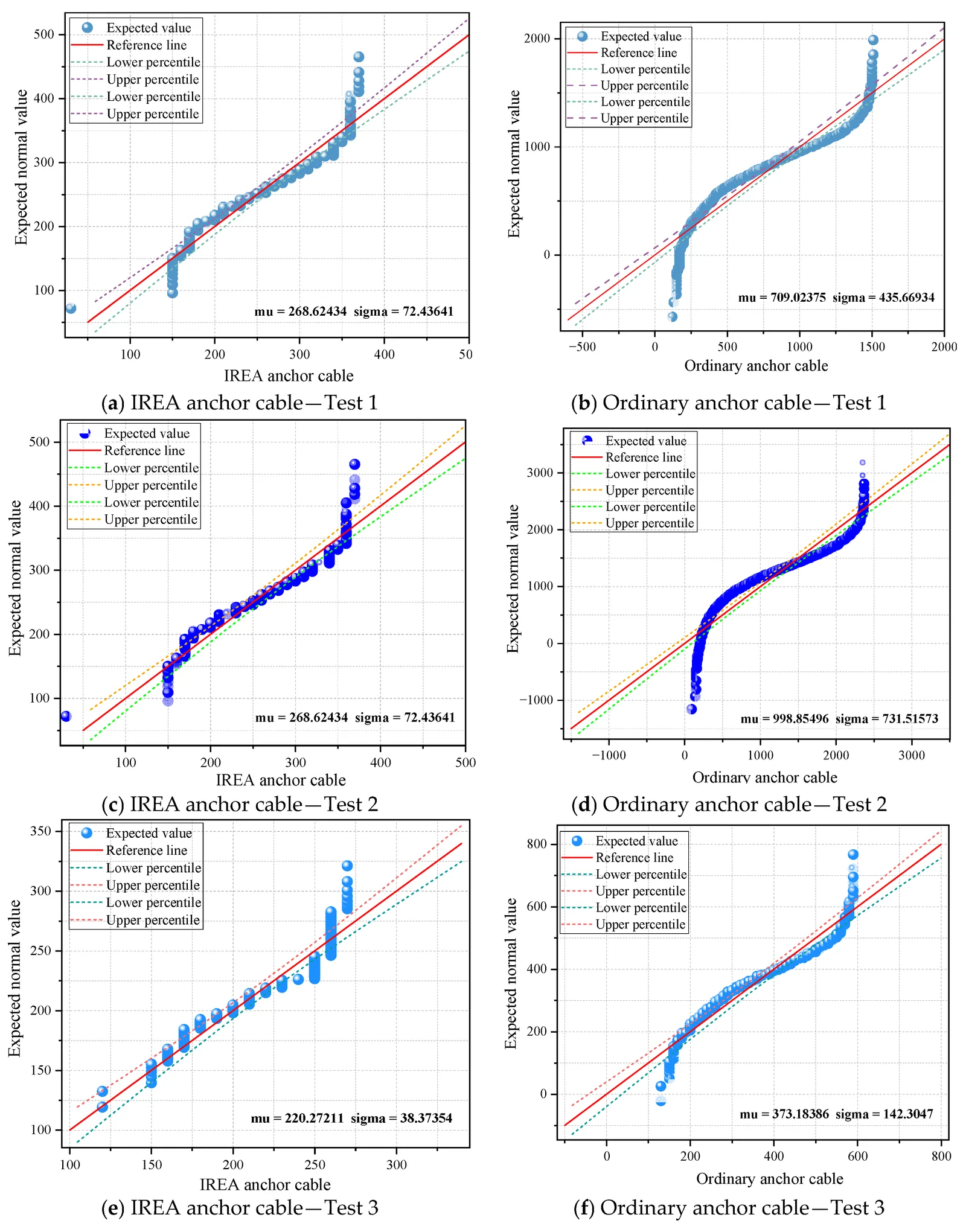
Q–Q inspection plots generated from three sets of experimental data for the ordinary anchor cable and IREA anchor cable.

**Figure 6:**
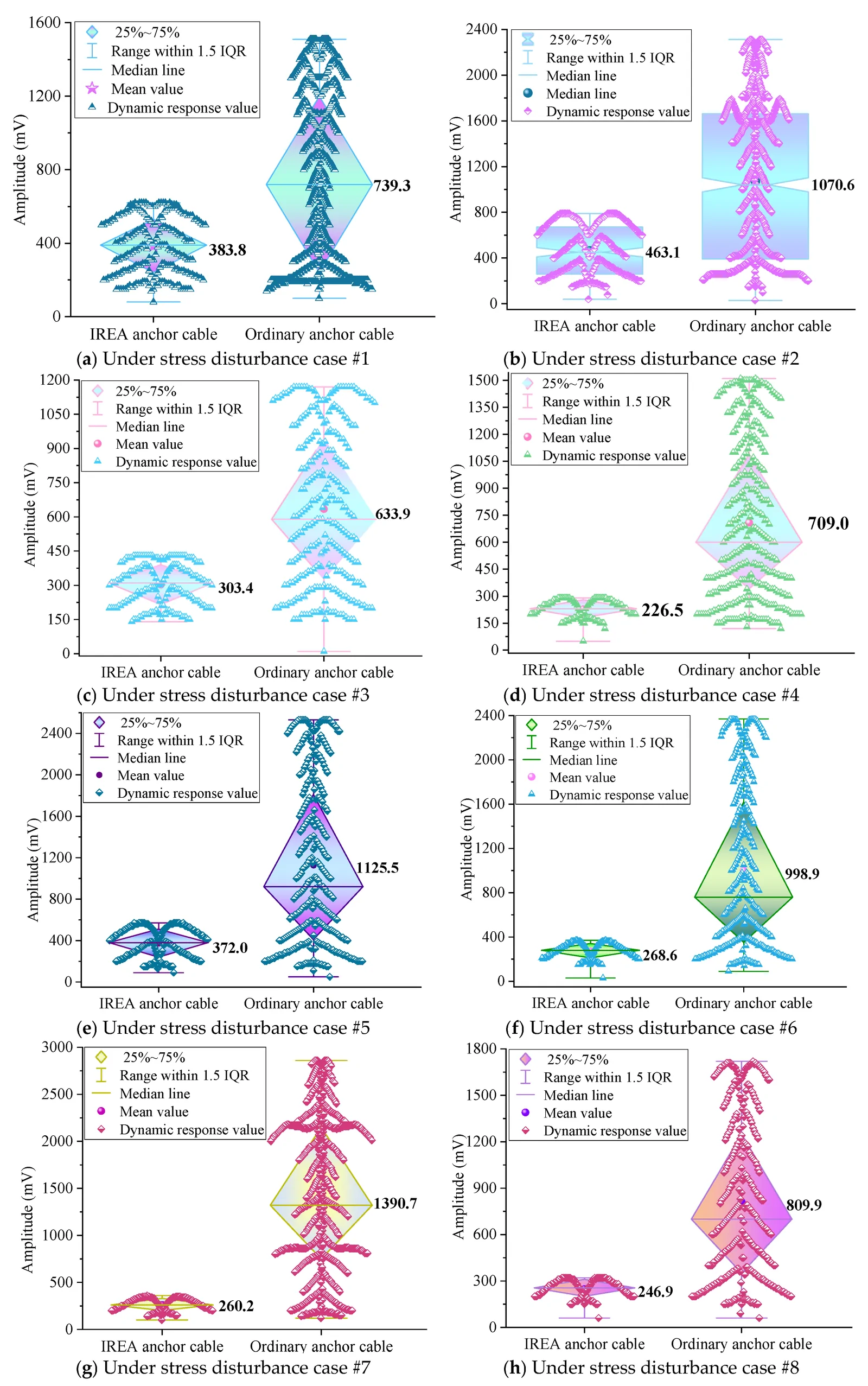
Box line chart comparison of IREA anchor cable and ordinary cable under stress disturbance cases #1~#8.

**Figure 7:**
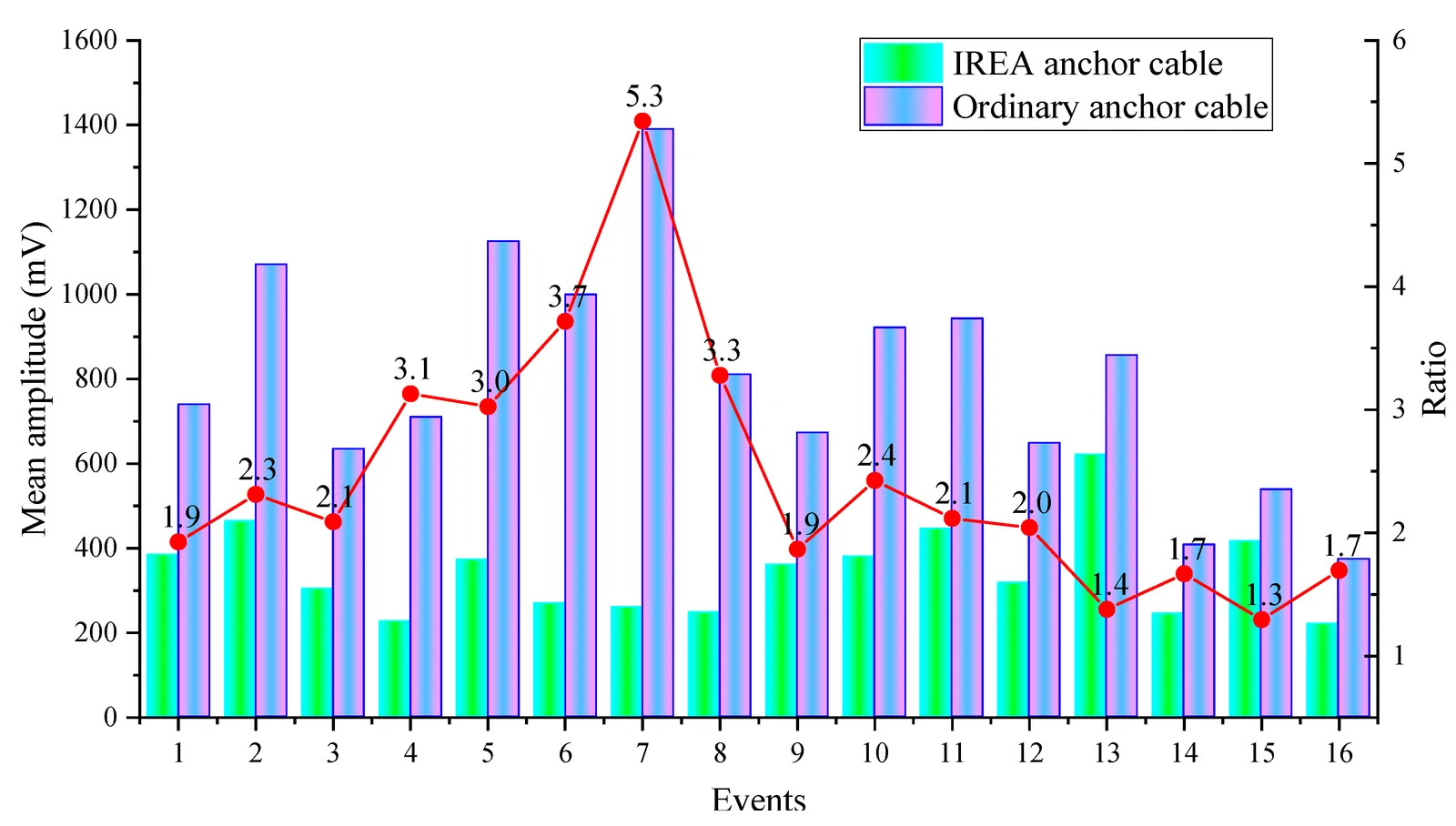
Response means amplitudes and ratios of two kinds of anchor cables under the influence of 16 disturbance cases.

**Figure 8:**
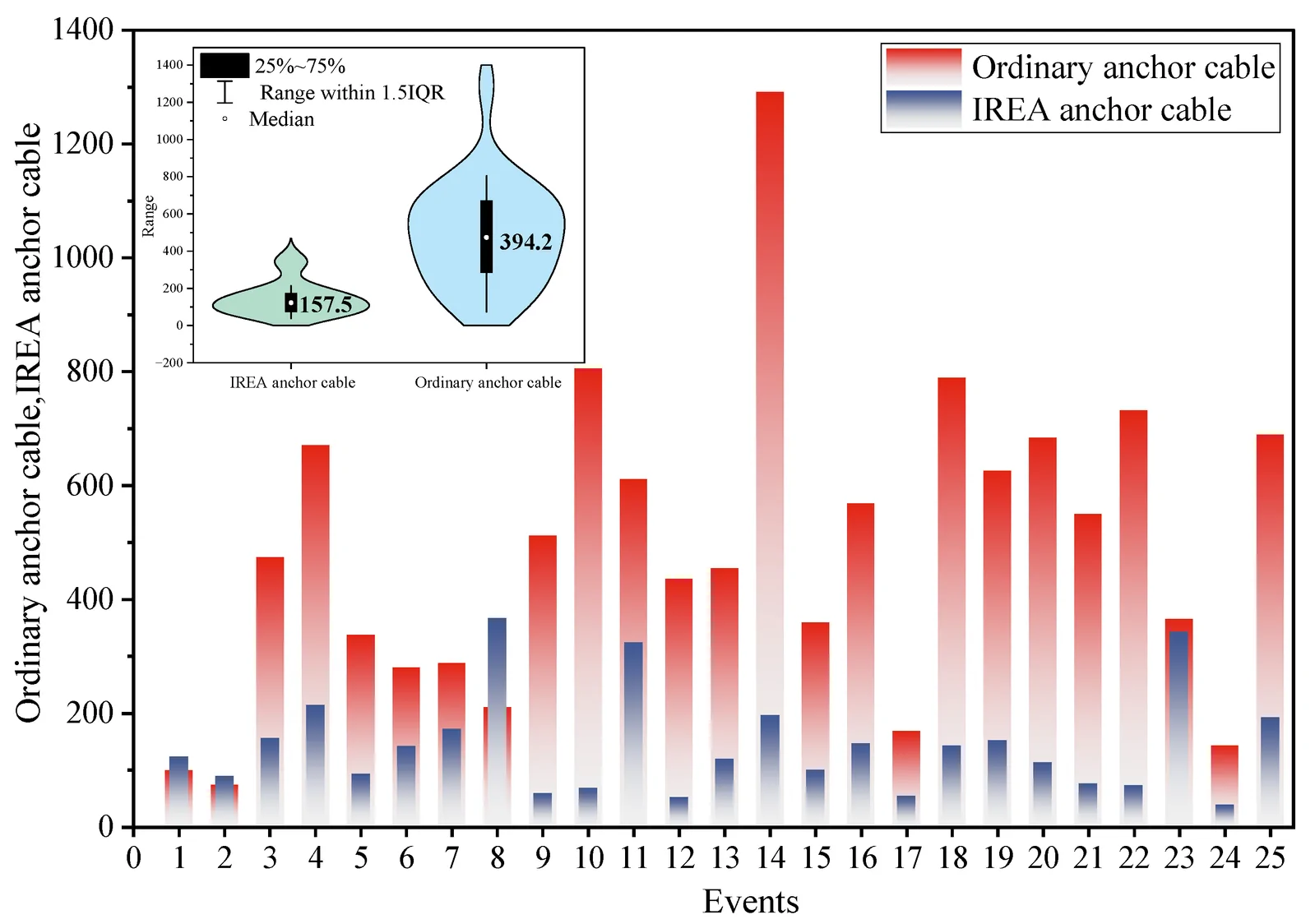
Standard deviation comparison between IREA anchor cable and ordinary anchor cable.

**Figure 9:**
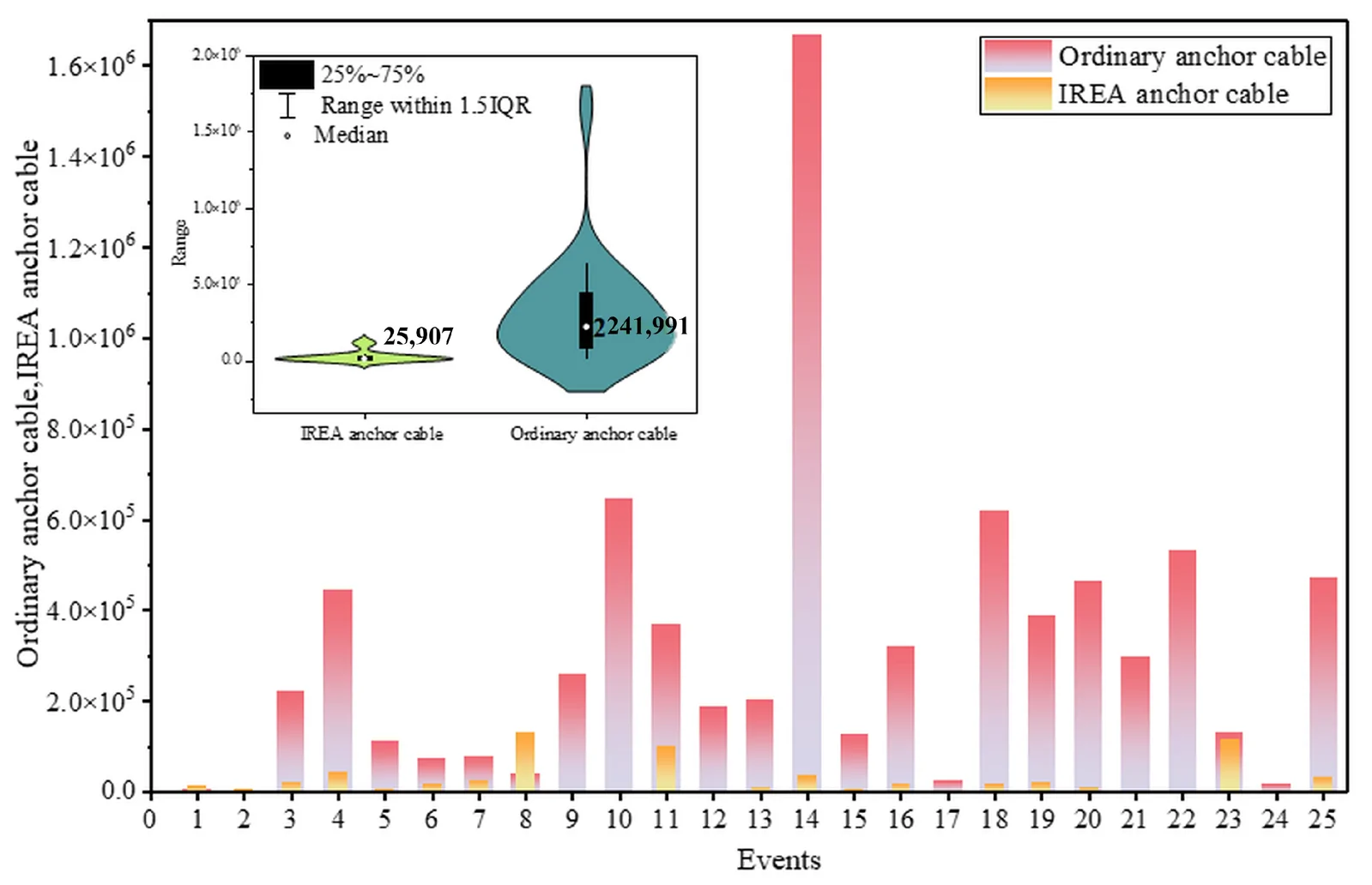
Variance comparison between IREA anchor cable and ordinary anchor cable.

**Figure 10:**
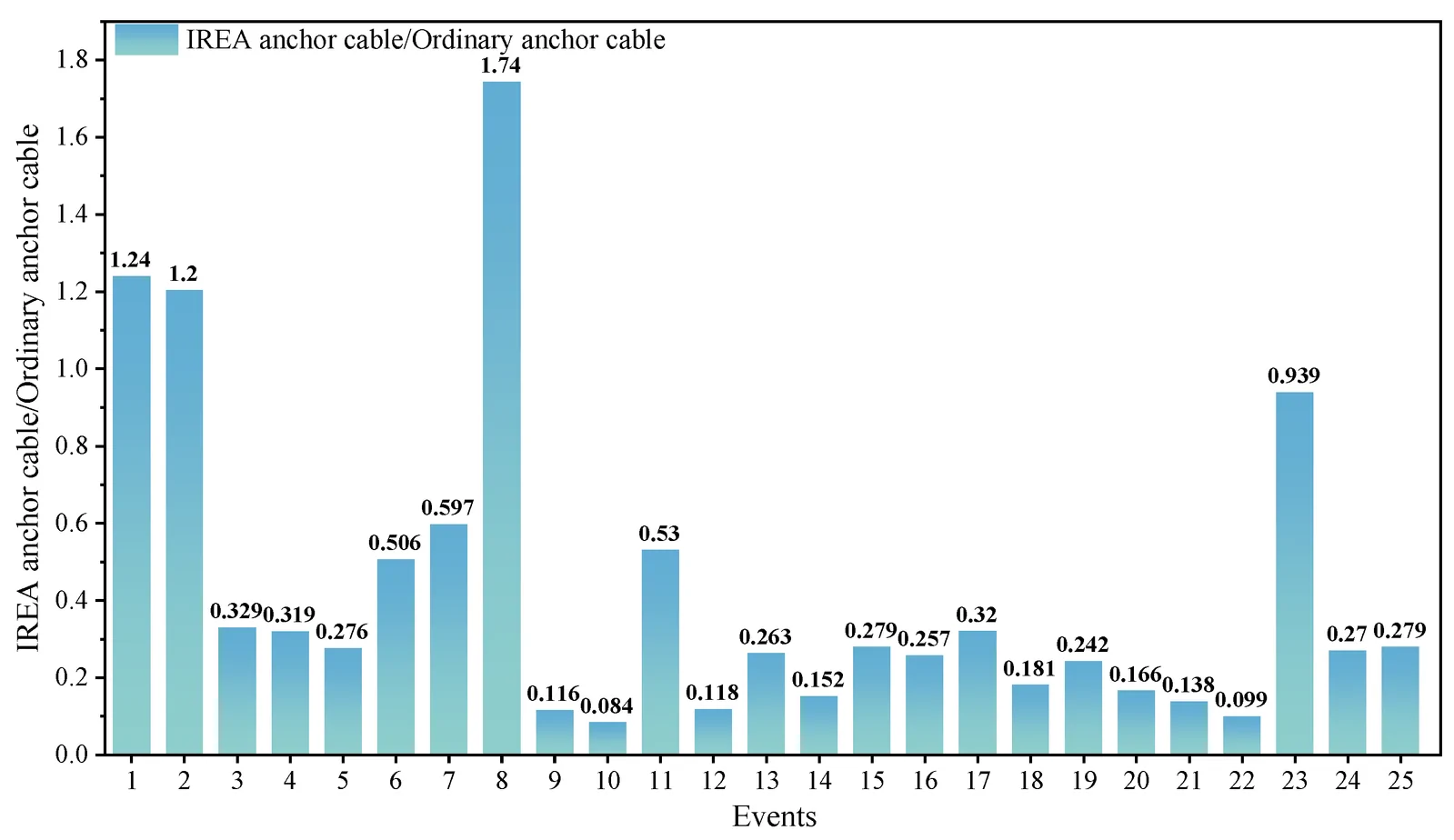
Ratios of standard deviation of IREA anchor cable/ordinary anchor cable.

**Figure 11:**
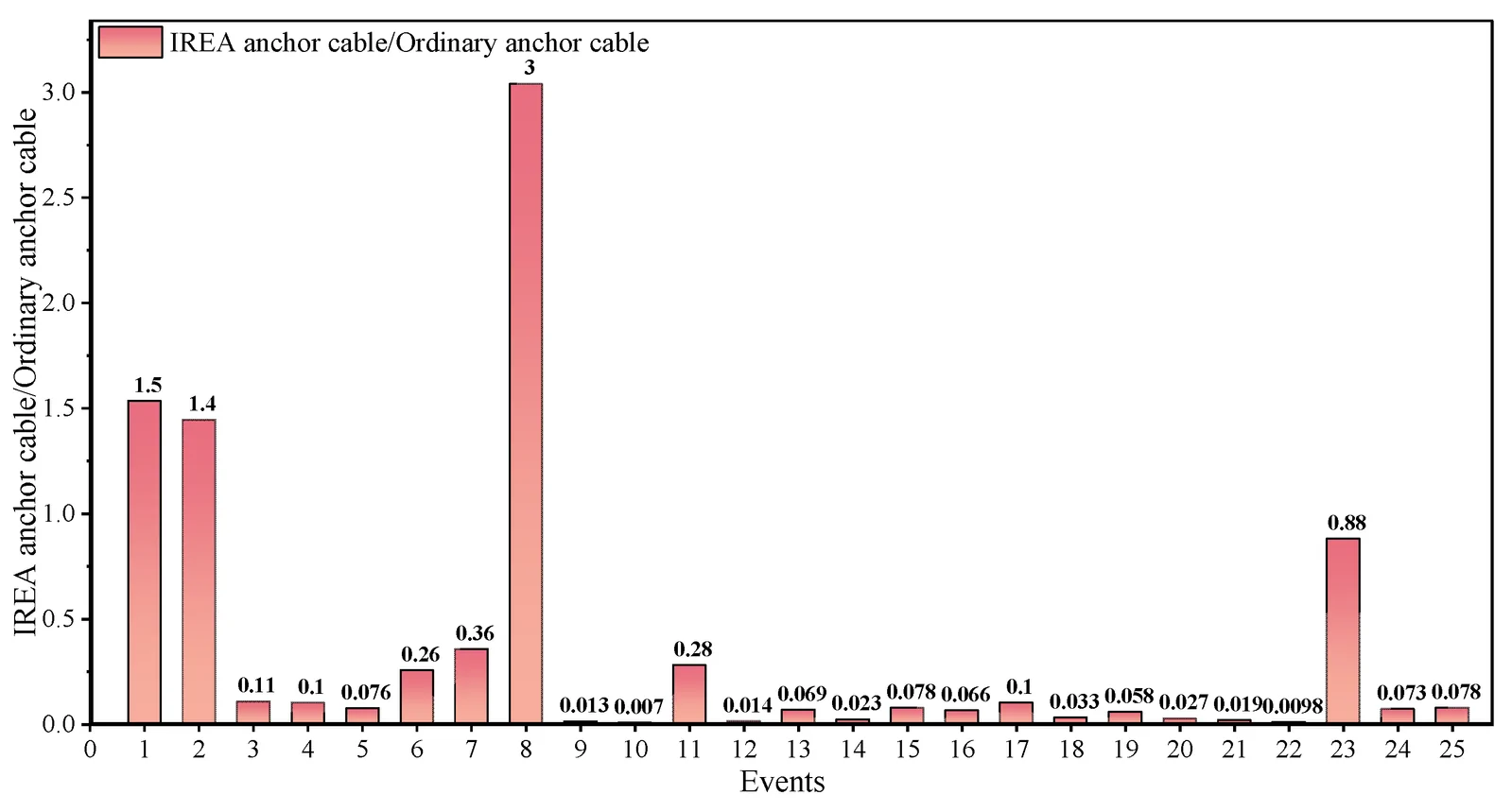
Ratios of variance of IREA anchor cable/ordinary anchor cable.

## Data Availability

The data presented in this study are available on request from the corresponding author.
